# Regional Variations in the Incidence of Lichen Sclerosus in Sweden: Insights from a Nationwide Register Study (2001–2021)

**DOI:** 10.3390/jcm13247836

**Published:** 2024-12-22

**Authors:** Sandra Jerkovic Gulin, Georgios Kravvas, Oliver Seifert

**Affiliations:** 1 Department of Dermatology and Venereology, Ryhov County Hospital, Sjukhusgatan, 553 05 Jönköping, Sweden; oliver.seifert@liu.se; 2 Division of Cell Biology, Department of Biomedical and Clinical Sciences, Faculty of Medicine and Health Sciences, Linköping University, 581 83 Linköping, Sweden; 3 Department of Dermatology, University College London Hospitals, 235 Euston Road, London NW1 2BU, UK; kravvas@hotmail.com

**Keywords:** lichen sclerosus, genital dermatoses, register study, epidemiology

## Abstract

**Background**: Lichen Sclerosus (LSc) is a chronic inflammatory skin condition predominantly affecting the anogenital regions, with a well-recognised potential for malignancy. This study examines the incidence, demographic characteristics, and regional distribution of LSc in Sweden over a 20-year period. The analysis is based on data from the Swedish National Patient Register (NPR), with a focus on cases diagnosed in specialist care settings. **Methods:** A nationwide register-based study was conducted using data from the NPR, identifying cases of LSc diagnosed between 1 January 2001 and 1 January 2021. Data analysis explored incidence by region, sex, age, and diagnostic care setting. A total of 154,424 patients with LSc were included, and the control group consisted of the general Swedish population without known LSc. **Results**: The mean annual incidence of LSc was 0.81 per 1000 individuals across Sweden, with higher rates in females (1.14 per 1000) compared to males (0.47 per 1000). Incidence varied significantly across regions, with Blekinge, Kalmar, and Gotland exhibiting the highest rates. This study analysed the distribution of LSc diagnoses across medical specialties, finding that 29.8% of cases were managed by dermatology and venereology, while 17.2% were handled by gynaecology and obstetrics. The analysis of marital status revealed that the proportion of married and divorced LSc patients was significantly lower than the national averages for men and women. **Conclusions**: This study highlights significant regional variations in LSc incidence. Future research should investigate whether environmental factors, genetic predisposition, socioeconomic disparities, or variations in healthcare access contribute to the variations in incidence. Such insights could lead to more targeted public health strategies for managing LSc across different regions.

## 1. Introduction

Lichen sclerosus (LSc) is a chronic inflammatory condition predominantly affecting the anogenital region. It may be asymptomatic or present with symptoms such as itching, burning, discoloration, or rashes. If left untreated, LSc can result in significant anatomical changes and functional impairment [1,2]. The disease affects both genders across all age groups, with females exhibiting two peak incidence periods, prepubertal years and postmenopausal age, while in males, it frequently presents in childhood or early adulthood [2,3,4,5]. The standard treatment includes super-potent topical corticosteroids (clobetasol propionate ointment) and in resistant cases among males, circumcision is considered [6]. The underlying aetiology of LSc is not fully understood, although several mechanisms have been implicated, including autoimmune processes, genetic background, hygiene, and alterations in the microbiome [7,8,9]. There is mounting evidence to suggest that chronic exposure to urine is key in the development of LSc. Genetic predisposition is also thought to play a role, as a positive family history can be elicited in approximately 12% of LSc cases [10].

A significant clinical concern in patients with LSc is the increased risk of malignancies, particularly genital squamous cell carcinoma (SCC), but also the increasing risk of melanoma [11,12,13,14]. Additionally, LSc is associated with a heightened risk of genital precancers, such as vulvar intraepithelial neoplasia (VIN) and penile intraepithelial neoplasia (PeIN) [14] An elevated incidence of other malignancies, including breast, bladder, and prostate cancers, has also been observed in individuals with LSc. A recent nationwide study in Sweden has highlighted significantly elevated odds ratios (OR) for vulvar SCC (OR = 8.3) and penile SCC (OR = 8.9), underscoring the need for vigilant monitoring and targeted therapeutic interventions for patients with LSc. Comorbid autoimmune and endocrine diseases such as thyroiditis, diabetes mellitus type 1, and vitiligo have also been frequently observed [15].

Despite growing recognition of LSc, epidemiological data remain limited, especially regarding regional and demographic distributions. In Sweden, the Swedish National Patient Register (NPR) offers a unique opportunity to study LSc epidemiology, although it only captures cases diagnosed in secondary specialist care settings. This study aims to address the gap in the literature by examining regional incidence, age and sex distribution, and marital status in LSc patients diagnosed in Sweden over a 20-year period.

## 2. Methods

### 2.1. Ethical Approval

This registry-based investigation employed data that had been rigorously pseudonymized in compliance with the General Data Protection Regulation (GDPR). Ethical clearance for this study was secured from the Swedish Ethical Review Authority on 15 November 2021, under file number 2021-05590-01.

### 2.2. Study Population and Data Source

This study is part of a larger nationwide register study conducted as a retrospective cohort analysis, utilizing data from the National Patient Register (NPR) in Sweden. Anonymized data were obtained for 154,424 individuals diagnosed with LSc (ICD-10: L90.0) between 1 January 2001 and 1 January 2021. The dataset included demographic and clinical information such as sex, age, year of diagnosis, and comorbidity status. After inclusion, patients were stratified by sex and age, with age divided into 10-year intervals (e.g., 0–9 years or 10–19 years, continuing up to 90+ years for individuals aged 90 and older).

The analysis was limited to LSc cases identified in secondary and tertiary (specialist) care settings, as the NPR in Sweden does not capture primary care diagnoses. This limitation meant that cases diagnosed solely in primary care were not available for the study.

To provide a robust comparison, the complete general Swedish population served as a control group. The mean annual population includes 9,371,975 individuals. Control subjects had no documented diagnosis of LSc, ensuring they represented a non-affected population. General population data, including sex, age, and year, were obtained from the Statistics Sweden database (www.scb.se, accessed on 9 April 2024). The case group’s age was defined by their age at initial LSc diagnosis, enabling age- and sex-specific incidence analysis.

### 2.3. Swedish National Patient Register

The Swedish National Patient Register (NPR), established in 1964 by the National Board of Health and Welfare, serves as an extensive resource for tracking nearly all inpatient healthcare services across public and private sectors in Sweden, excluding primary care. Consequently, individuals diagnosed with LSc solely in primary care are not recorded in the NPR and could therefore not be included in this study. The NPR collects a range of data, including patient demographic details (personal identification number, sex, age, and county of residence), administrative records, hospital identifiers, and clinical diagnoses.

Since 1997, diagnoses within the NPR have adhered to the ICD-10 coding system. Inpatient data coverage within the NPR is close to complete, while outpatient data coverage is approximately 87%. In 2001, outpatient reporting became mandatory for the NPR, although primary care data remain excluded from this register [16].

### 2.4. Statistical Analysis

Incidence rates were calculated as the number of LSc cases per 1000 individuals per year, stratified by sex, age group, and region. These rates were calculated annually and separately by gender. The annual average population for each of Sweden’s 21 regions was obtained from SCB and used to analyse the incidence rate for each region. For statistical precision, 95% confidence intervals (CIs) were calculated using the Poisson distribution for each incidence estimate. The z-test was used to assess whether the difference in incidence rates between the regions is statistically significant. Statistical analyses were performed using IBM SPSS version 28.0. A *p*-value below 0.05 was considered statistically significant.

The annual marital status for the entire Swedish population from 2001 to 2021 was obtained from SCB to compare with the marital status of LSc cases. To account for age differences between these populations, age-adjusted rates were calculated. In brief, the national population served as the standard population. Age-specific rates were calculated for each age group within each population, then multiplied by the proportion of that age group in the standard population. The resulting weighted rates were then summed to produce the age-adjusted rate for each population. Then, 95% confidence intervals (CIs) were calculated using the Poisson distribution to compare the marital status between the national population and LSc cases.

## 3. Results

### 3.1. Incidence by Region

The estimated annual incidence of LSc was 0.81 cases per 1000 individuals, based on a total population of 9,371,975.

**Figure 1:**
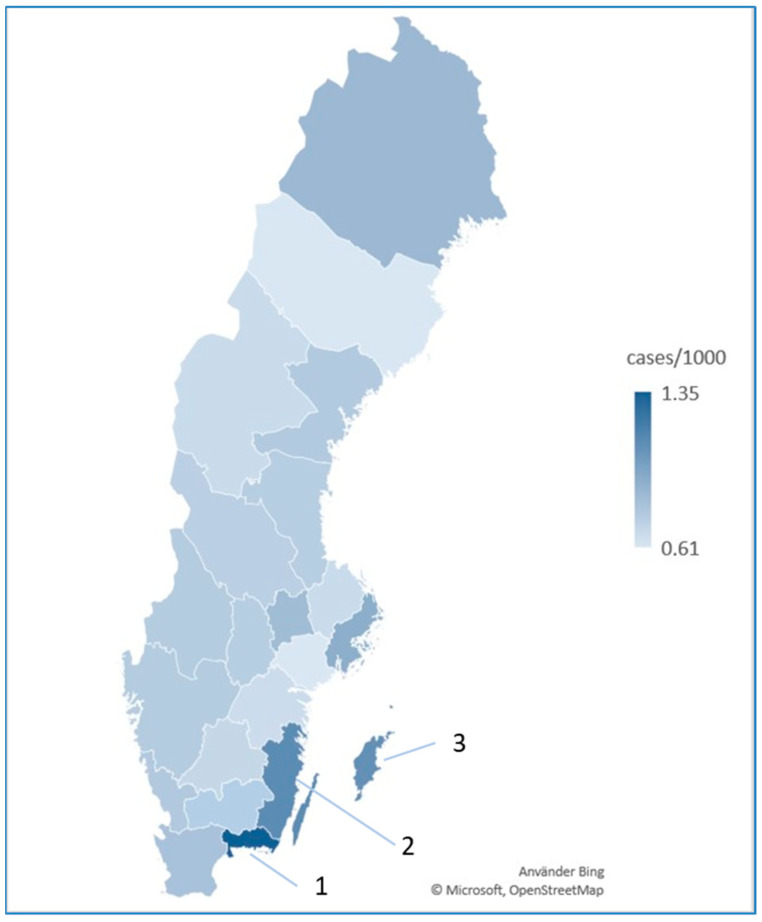
The incidence of LSc in Sweden. Map depicting regional variations in the incidence of LSc across Swedish counties: darker blue shades represent higher incidences, while lighter blue shades represent lower incidences. The highest incidence rates were observed in the southeastern counties of (1) Blekinge (2.02 per 1000), (2) Kalmar (1.61 per 1000), and (3) Gotland (1.68 per 1000).

Regionally, the highest incidence rates were observed in the southeastern counties of Blekinge (2.02 per 1000), Gotland (1.68 per 1000), and Kalmar (1.61 per 1000). In contrast, Västerbotten and Södermanland had lower rates, both at 0.61 per 1000, and Jämtland and Västerbotten reported 0.94 and 0.85 per 1000, respectively (Table 1, Figure 1).

Incidence rates of LSc showed regional and gender-specific differences, with consistently higher rates among females in all regions. Southeastern counties such as Blekinge, Kalmar, and Gotland reported the highest incidence, with female prevalence nearing 2 per 1000 individuals in Blekinge. Conversely, Västerbotten and Jämtland had the lowest rates, with female incidence below 1 per 1000.

**Figure 2:**
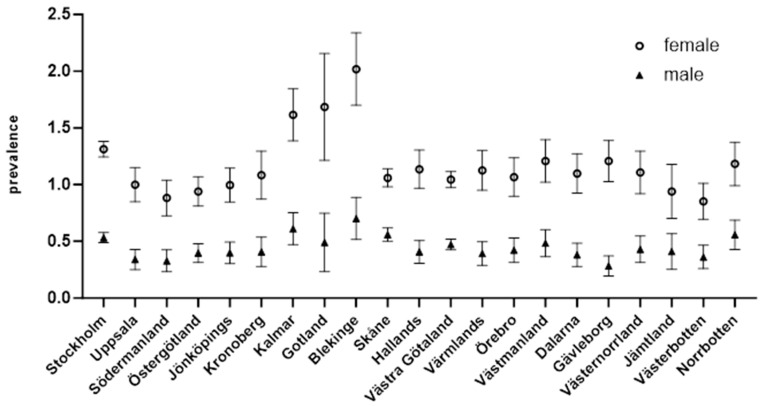
Annual mean incidence of LSc per 1000 inhabitants in Swedish counties, stratified by gender.

Figure 2 illustrates the annual mean incidence of LSc across Swedish counties, highlighting regional and gender-specific variations. Incidence rates are consistently higher among females compared to males in all regions. Southeastern counties, including Blekinge, Kalmar, and Gotland, exhibit the highest incidence rates, with female prevalence approaching 2 per 1000 individuals in Blekinge. In contrast, counties such as Västerbotten and Jämtland report some of the lowest incidence rates, with female incidence below 1 per 1000. Error bars represent 95% confidence intervals, indicating more precise estimates in regions with higher incidence rates. The data underscore significant gender disparities and a regional clustering of elevated LSc incidence in southeastern Sweden.

Table 2 presents pairwise comparisons of LSc incidence rates across Swedish counties, indicating significant differences at a 95% confidence level. Stockholm has significant differences in incidence with most other counties, except for Gotland, Västmanland, and Norrbotten, where no significant difference (ns) was observed. Blekinge, known for its high incidence, shows significant differences with nearly all other counties, emphasizing its unique prevalence profile. This analysis underscores the substantial geographic variability in LSc prevalence, with southeastern counties like Blekinge having significantly higher rates compared to other regions. These findings may inform targeted resource allocation for LSc management in high-incidence areas.

### 3.2. Distribution of LSc Diagnoses by Medical Speciality

The majority of cases were diagnosed in Dermatology and Venereology (29.8%, 45,253 cases), followed by Gynaecology and Obstetrics (17.2%, 26,029 cases), highlighting these as the primary specialities managing LSc. Other significant contributors included General Surgery (9.5%, 14,358 cases) and Plastic Surgery (8.3%, 12,571 cases), likely reflecting cases that require surgical or reconstructive intervention. Less commonly involved specialities included Internal Medicine (3.8%, 5711 cases), Otorhinolaryngology (3.3%, 4997 cases), Paediatrics (1.8%, 2777 cases), and Urology (11.4%, 17,303 cases). Smaller proportions were also registered by Ophthalmology (2.7%, 4040 cases), Infectious Diseases (1.2%, 1836 cases), Rheumatology (0.9%, 1322 cases), and Oncology (0.9%, 1349 cases). The “Other” category, covering various non-specified specialties, accounts for 6.3% (9589 cases).

**Figure 3:**
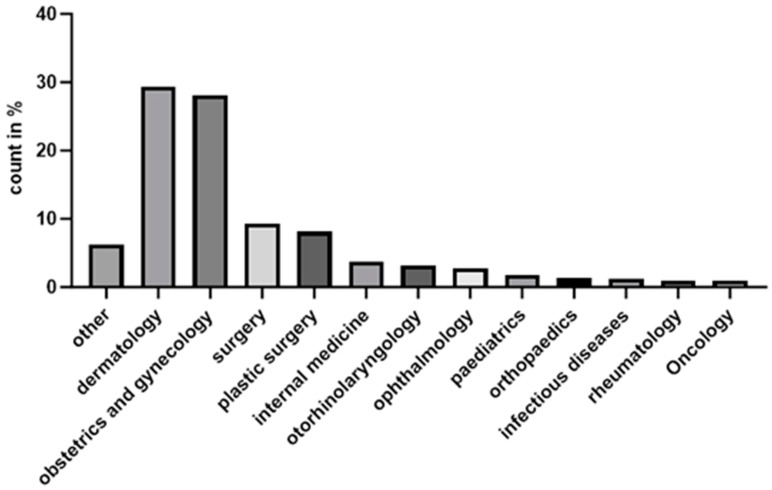
Distribution of LSc diagnoses by medical specialty.

The table presents the distribution of LSc diagnoses across different medical specialties in outpatient care, based on data from 154,424 registered cases. The majority of diagnoses were made in dermatology and venereology (Figure 3), followed by gynaecology and obstetrics, which accounted for 17.2% of cases (26,029 diagnoses).

### 3.3. Marital Status

**Figure 4:**
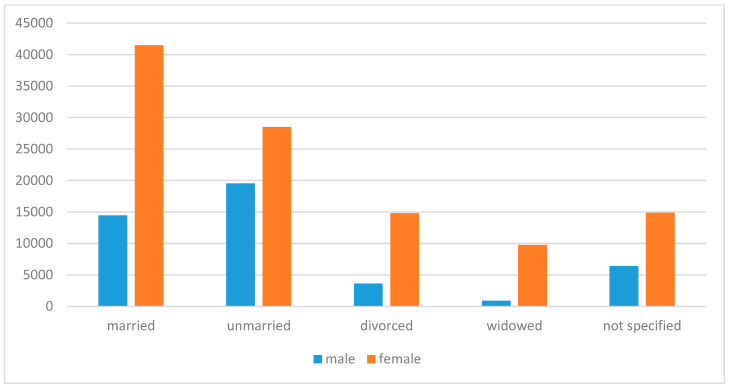
Marital status distribution among LSc patients by gender. The figure displays the proportion of married and divorced individuals in the LSc cohort, stratified by gender, alongside corresponding rates in the general population.

This study examined the distribution of marital status among individuals diagnosed with LSc compared to the general Swedish population. Figure 4 illustrates the overall marital status, stratified by gender.

Among LSc cases, 14,472 married men and 41,461 married women were identified, alongside 3617 divorced men and 14,801 divorced women. In contrast, data from Statistics Sweden (2001–2020) indicate that the mean annual married population without LSc consists of 1,600,019 men and 1,568,376 women, while the divorced population includes 392,237 men and 495,769 women.

A comparison of marital status rates revealed differences between the LSc cohort and the general population. In the LSc cohort, the crude rate of married men was 32.2%, with an age-adjusted rate of 29.7% (95% CI: 29.2–30.1%). For women with LSc, the crude married rate was 37.9%, and the age-adjusted rate was 29.6% (95% CI: 29.3–30.1%). By contrast, in the general population (excluding LSc cases), the married rates were 33.9% for men (95% CI: 33.9–34.0%) and 33.6% for women (95% CI: 33.5–33.7%). These findings suggest a significantly lower proportion of married individuals among LSc patients after adjusting for age.

Divorce rates also differed between the LSc cohort and the general population. In the LSc cohort, the crude divorce rate for men was 8.0%, with an age-adjusted rate of 7.6% (95% CI: 7.3–7.8%). For women with LSc, the crude divorce rate was 13.5%, and the age-adjusted rate was 10.2% (95% CI: 9.9–10.3%). In comparison, the divorce rates in the general population were 8.3% for men (95% CI: 8.3–8.4%) and 10.6% for women (95% CI: 10.6–10.7%). These results highlight a significantly lower proportion of divorced individuals among LSc patients following age adjustment.

## 4. Discussion

This study provides novel insights into the geographic and demographic distribution of genital LSc in Sweden over a 20-year period, highlighting notable regional and gender-specific variations in incidence. Our data reveal clear regional differences, with certain southeastern counties, such as Blekinge, Kalmar, and Gotland, reporting significantly higher incidence rates. This regional variability in LSc incidence may be influenced by factors including population demographics, healthcare accessibility, and local diagnostic practices. The elevated incidence in southeastern regions could reflect differences in healthcare-seeking behaviour or diagnostic frequency, possibly indicating more active case identification in these areas.

The consistently higher incidence among females across all regions aligns with the well-documented gender disparity in LSc, which may be due to biological factors or greater clinical awareness and diagnosis of LSc in females, particularly in postmenopausal women who are more commonly affected. This gender difference underscores the need for tailored approaches to screening and diagnosis that consider the specific vulnerabilities of female populations [6,17,18].

These findings carry practical implications for healthcare planning and resource allocation. Counties with higher LSc incidence, especially Blekinge, Kalmar, and Gotland, would benefit from targeted interventions and increased resources to manage the greater burden of disease. Directing healthcare resources to these high-prevalence regions could enhance patient outcomes and support better LSc management. Such targeted resource allocation is particularly important in areas where patients may encounter barriers to diagnosis and treatment, ensuring more equitable healthcare delivery across the country.

There is a substantial body of literature collectively indicating that social, environmental, and behavioural factors play a pivotal role in shaping the geographic distribution of diseases [19]. However, this does not rule out the potential influence of genetic factors. The higher incidence of LSc in certain regions may be partially explained by genetic predispositions [9,10,20,21,22,23]. LSc is associated with an increased prevalence of other autoimmune conditions, such as thyroiditis and type 1 diabetes, suggesting shared genetic susceptibility [23]. Specific HLA alleles, such as HLA-DQ7 and HLA-DR12, have been linked to LSc in previous studies [8,24,25]. Regions with a higher prevalence of these alleles due to population genetics may exhibit elevated LSc incidence. Historical patterns of migration and settlement can lead to the concentration of particular genetic traits in certain regions. In areas like Blekinge and Gotland, which have historically had relatively isolated populations, founder effects may have amplified the prevalence of genetic risk factors for LSc. This phenomenon occurs when a small initial population carries specific alleles that become widespread in their descendants. Regional differences could also be explained by the familial clustering of LSc. Families within specific areas might share genetic predispositions, increasing local incidence. This clustering effect could be more pronounced in regions with limited genetic diversity or smaller, tightly knit populations [20,21,26,27,28]. Beyond genetic predispositions, epigenetic changes may affect how genetic risks manifest. Environmental differences between regions, such as exposure to infections or dietary habits, could drive these epigenetic modifications, exacerbating genetic risk factors in certain populations [29]. To explore these hypotheses, genetic sequencing and population-level studies in high-incidence areas such as Blekinge, Kalmar, and Gotland are needed. These investigations could identify specific genetic markers contributing to LSc susceptibility, helping to explain the observed geographic patterns.

A significant limitation of this study is the absence of primary care data in the Swedish National Patient Register (NPR), as many LSc cases are likely diagnosed and managed in primary care settings without referral to specialists. Consequently, the true incidence of LSc in Sweden is likely underestimated and may be considerably higher than reported.

Regarding sex, the incidence of LSc was particularly elevated among postmenopausal women and those aged 50–59, suggesting a potential role of hormonal factors in disease onset and progression [5]. Additionally, in men, phimosis—commonly associated with LSc—is often diagnosed and coded as phimosis rather than LSc by urologists and primary care providers. Furthermore, tissue excised during circumcision is rarely sent for histopathological examination, contributing to underreported prevalence rates of LSc. Increased awareness about the relationship between phimosis and LSc, as well as LSc in general, is necessary to improve diagnostic accuracy and reporting.

These factors may also explain why women exhibited a significantly higher incidence of LSc (1.14 per 1000) compared to men (0.47 per 1000) in this study. Many men who undergo circumcision for phimosis may not have their LSc diagnosis recorded in the NPR, leading to an underestimation of the true incidence in males.

This study analysed 154,424 outpatient cases of LSc to understand its distribution across medical specialties. Dermatology and venereology (29.8%) and gynaecology and obstetrics (17.2%) accounted for most diagnoses, reflecting LSc’s dermatological and genital manifestations. Urology (11.4%) and surgical fields, including general surgery (9.5%) and plastic surgery (8.3%), highlight LSc’s management in cases involving scarring or advanced disease. Other specialties, such as internal medicine, paediatrics, and ophthalmology also reported diagnosis of LSc, but of course in smaller proportions, likely due to patient medical history or documentation from primary care.

These findings underline the need for interdisciplinary collaboration, particularly among dermatologists, gynaecologists, and urologists, to ensure timely diagnosis and comprehensive management. Targeted training and resources in high-prevalence specialties could improve outcomes. Furthermore, the observed distribution suggests a role for surgical fields in managing complications.

This study also examined marital status, finding a lower proportion of married and divorced individuals among LSc cases compared to the general population. This difference persisted after age adjustment and may indicate unique socio-demographic characteristics or barriers to diagnosis among divorced individuals. Chronic conditions like LSc may impact social and marital relationships, similar to other chronic skin diseases [30]. Future research should explore the social and psychological impacts of LSc, its influence on marital stability, and potential socio-economic factors affecting diagnosis and care.

## Figures and Tables

**Table 1:** The incidence of LSc in Sweden. Table showing the annual incidence of LSc across all Swedish counties: data presented by county with corresponding incidence rates.

Swedish County	Incidence
**Stockholm**	0.93 (0.88–0.97)
**Uppsala**	0.67 (0.58–0.76)
**Södermanland**	0.61 (0.51–0.70)
**Östergötland**	0.67 (0.59–0.74)
**Jönköping**	0.70 (0.61–0.78)
**Kronoberg**	0.74 (0.62–0.86)
**Kalmar**	1.11 (0.98–1.15)
**Gotland**	1.09 (0.82–1.36)
**Blekinge**	1.35 (1.17–1.53)
**Skåne**	0.81 (0.76–0.86)
**Halland**	0.77 (0.67–0.87)
**Västra Götaland**	0.76 (0.72–0.80)
**Värmland**	0.76 (0.66–0.86)
**Örebro**	0.74 (0.64–0.84)
**Västmanland**	0.85 (0.73–0.96)
**Dalarna**	0.74 (0.64–0.84)
**Gävleborg**	0.74 (0.64–0.85)
**Västernorrland**	0.77 (0.66–0.88)
**Jämtland**	0.67 (0.53–0.82)
**Västerbotten**	0.61 (0.51–0.70)
**Norrbotten**	0.86 (0.75–0.98)

**Table 2:** Pairwise comparison of LSc incidence rates across Swedish counties. This table presents a pairwise comparison of LSc incidence rates across Swedish counties, with statistical significance assessed at the 95% confidence level. Counties marked with a “*” indicate significantly different incidence rates, highlighting notable regional variation. Comparisons labelled as “ns” denote non-significant differences, suggesting similar LSc incidence between these counties. These results emphasize regions with distinct versus comparable LSc incidence patterns.

	1	3	4	5	6	7	8	9	10	12	13	14	17	18	19	20	21	22	23	24	25
Stockholm (1)		*	*	*	*	*	*	ns	*	*	*	*	*	*	ns	*	*	*	*	*	ns
Uppsala (3)			ns	ns	ns	ns	*	*	*	*	ns	ns	ns	ns	ns	ns	ns	ns	ns	ns	*
Södermanland (4)				ns	ns	ns	*	*	*	*	ns	*	ns	ns	*	ns	ns	ns	ns	ns	*
Östergötland (5)					ns	ns	*	*	*	*	ns	ns	ns	ns	*	ns	ns	ns	ns	ns	*
Jönköping (6)						ns	*	*	*	*	ns	ns	ns	ns	ns	ns	ns	ns	ns	ns	*
Kronoberg (7)							*	*	*	ns	ns	ns	ns	ns	ns	ns	ns	ns	ns	ns	ns
Kalmar (8)								ns	ns	*	*	*	*	*	*	*	*	*	*	*	*
Gotland (9)									ns	*	*	*	*	*	ns	*	*	*	*	*	ns
Blekinge (10)										*	*	*	*	*	*	*	*	*	*	*	*
Skåne (12)											ns	ns	ns	ns	ns	ns	ns	ns	ns	*	ns
Halland (13)												ns	ns	ns	ns	ns	ns	ns	ns	*	ns
Västra Götaland (14)													ns	ns	ns	ns	ns	ns	ns	*	ns
Värmland (17)														ns	ns	ns	ns	ns	ns	*	ns
Örebro (18)																				*	ns
Västmanland (19)																				*	ns
Dalarna (20)																				ns	ns
Gävleborg (21)																				*	ns
Västernorrland (22)																				*	ns
Jämtland (23)																				ns	ns
Västerbotten (24)																					*
Norrbotten (25)																					

## Data Availability

Data and materials can be assessed by contacting one of the authors.
